# Two-test algorithms for infectious disease diagnosis: Implications for COVID-19

**DOI:** 10.1371/journal.pgph.0000293

**Published:** 2022-03-31

**Authors:** Sunil Pokharel, Lisa J. White, Jilian A. Sacks, Camille Escadafal, Amy Toporowski, Sahra Isse Mohammed, Solomon Chane Abera, Kekeletso Kao, Marcela De Melo Freitas, Sabine Dittrich

**Affiliations:** 1 Nuffield Department of Medicine, Centre for Tropical Medicine and Global Health, University of Oxford, Oxford, United Kingdom; 2 Foundation for Innovative New Diagnostics (FIND), Geneva, Switzerland; 3 Big Data Institute, Li Ka Shing Centre for Health Information and Discovery, Nuffield Department of Medicine, University of Oxford, Oxford, United Kingdom; 4 National Reference Laboratory, Ministry of Health, Mogadishu, Somalia; 5 World Health Organization Country Office in Somalia, Mogadishu, Somalia; University of Sao Paulo: Universidade de Sao Paulo, BRAZIL

## Abstract

Diagnostic assays for various infectious diseases, including COVID-19, have been challenged for their utility as standalone point-of-care diagnostic tests due to suboptimal accuracy, complexity, high cost or long turnaround times for results. It is therefore critical to optimise their use to meet the needs of users. We used a simulation approach to estimate diagnostic outcomes, number of tests required and average turnaround time of using two-test algorithms compared with singular testing; the two tests were reverse transcription polymerase chain reaction (RT-PCR) and an antigen-based rapid diagnostic test (Ag-RDT). A web-based application of the model was developed to visualise and compare diagnostic outcomes for different disease prevalence and test performance characteristics (sensitivity and specificity). We tested the model using hypothetical prevalence data for COVID-19, representing low- and high-prevalence contexts and performance characteristics of RT-PCR and Ag-RDTs. The two-test algorithm when RT-PCR was applied to samples negative by Ag-RDT predicted gains in sensitivity of 27% and 7%, respectively, compared with Ag-RDT and RT-PCR alone. Similarly, when RT-PCR was applied to samples positive by Ag-RDT, specificity gains of 2.9% and 1.9%, respectively, were predicted. The algorithm using Ag-RDT followed by RT-PCR as a confirmatory test for positive patients limited the requirement of RT-PCR testing resources to 16,400 and 3,034 tests when testing a population of 100,000 with an infection prevalence of 20% and 0.05%, respectively. A two-test algorithm comprising a rapid screening test followed by confirmatory laboratory testing can reduce false positive rate, produce rapid results and conserve laboratory resources, but can lead to large number of missed cases in high prevalence setting. The web application of the model can identify the best testing strategies, tailored to specific use cases and we also present some examples how it was used as part of the Access to Covid-19 Tools (ACT) Accelerator Diagnostics Pillar.

## Introduction

The accurate and timely diagnosis of numerous infectious diseases is constrained by a lack of optimal diagnostic infrastructure and expertise, particularly at the point-of-care in low- and middle-income countries (LMICs) [1]. Rapid diagnostic tests (RDTs) provide a platform that is simple to use, cost effective and can provide rapid results; the use of malaria RDTs in malaria endemic settings has been shown to improve health outcomes [2]. However, outside of the widely used malaria RDT, available RDTs for many infectious diseases are limited by suboptimal accuracies and hence would benefit from being used as part of clearly defined diagnostic algorithms [3].

Diagnostic tests and their ability to detect a pathogen directly (antigenic or genomic material) or to detect infections by identifying the host response (antibody) have often formed part of diagnostic algorithms, where any decision to apply further tests is based on prior test results [4, 5]. This ‘two-test algorithm’ approach to diagnosis has been successfully adopted for HIV and hepatitis B and C [4, 5]. The currently recommended HIV testing algorithm includes a combination of two or three reactive tests, depending on the background infection prevalence [6]. The diagnostic algorithms currently used for hepatitis B virus (HBV) and hepatitis C virus (HCV) require a similar two-test approach, using either an enzyme immunoassay (EIA) or an RDT for the serological detection of hepatitis B surface antigen (HBsAg) or anti-HCV antibodies, with any positive serological results confirmed by viral detection using more expensive tests, such as nucleic acid testing [4, 7].

Reverse transcription polymerase chain reaction (RT-PCR) offers high accuracy and remains the ‘gold standard’ for the diagnosis of COVID-19. However, its utility for universal screening and diagnosis is limited by its complexity, cost and turnaround time; it is therefore inaccessible to a large proportion of the global population who live in resource-poor settings [8]. RDTs to detect antigens of SARS-CoV-2, the virus that causes COVID-19, have been developed and are currently being evaluated, but are less accurate compared with the accuracy of RT-PCR [9–11]. A recent Cochrane review estimated that the sensitivity of antigen RDTs (Ag-RDTs) for COVID-19 is 72% (95% CI 64.7%–79%) in symptomatic patients and 58.1% (95% CI 40.2–74.1) in asymptomatic patients compared with RT-PCR tests [8]. Nevertheless, these RDTs are used at border screening points, for screening asymptomatic individuals who have been in contact with a case, and for mass population screening programmes [12, 13].

The World Health Organization (WHO) recommends the use of Ag-RDTs for SARS-CoV-2 with >80% sensitivity and >97% specificity compared with RT-PCR for individuals who are symptomatic, meet the suspected case definition and/or are in a setting with a suspected outbreak, where the reference test (RT-PCR) is not available or has limited usefulness due to long turnaround times [14]. Nevertheless, confirmatory testing of an Ag-RDT result with RT-PCR should be performed wherever possible and a cautious interpretation of the result where confirmatory testing is not feasible is recommended. To optimise testing algorithms for different scenarios, it is critical to consider the trade-offs between the good accuracy but high costs and turnaround times of RT-PCR and the low costs and turnaround times but suboptimal accuracy of Ag-RDTs.

Sequential testing algorithms for multiple causes of acute febrile illnesses using a diagnostic test for each aetiology have been explored previously [3]. The implementation of adaptive algorithms based on local epidemiology (disease prevalence) was found to predict better diagnosis and add value to available tests. In the current study, we build on this approach to compare the impact of two-test algorithms versus singular testing for COVID-19-testing use cases. We tested the diagnostic abilities of Ag-RDTs and RT-PCR when these tests were combined in a two-test algorithm and compared this with singular testing. Comparisons of diagnostic outcomes (true positives and true negatives) were made when the tests were applied in a high-prevalence scenario, akin to diagnostic testing of symptomatic cases in hospital settings, and a low-prevalence scenario, to represent point-of-entry or border screening for infections. We then compared the average turnaround times to receive results and the demand for tests for each of the testing strategies. A web-based application was also developed, which can help to optimise testing strategies in different use cases, depending on the expected testing demand, prevalence of infection in the population and available resources.

## Methods

### Definitions

Singular testing means either one of the two tests is applied alone as the only diagnostic tool for screening and/or diagnostic purposes. This approach is adopted when an optimal diagnostic test is available at the point-of-care, for example, the malaria RDT, which is adopted as a singular test for the diagnosis of malaria [15]. In the present study, singular testing serves as a comparator.

Combination testing involves two available tests being applied in either a sequential or a simultaneous testing algorithm, as described below. A combination testing strategy is used when the combination enhances the value of the tests in terms of clinical diagnosis and screening. Combination testing has previously been adopted for various infectious diseases, including HIV, HBV and HCV [4, 5].

Sequential testing is considered to be more appropriate than simultaneous testing in terms of the demand for tests, logistics and resources required for its implementation and is preferred in practice; therefore, we have focussed on this approach. Nonetheless, the results of diagnostic outcomes (true and false positives and true and false negatives) described below also hold true for the corresponding simultaneous testing strategies.

### Sequential testing

**Fig 1:**
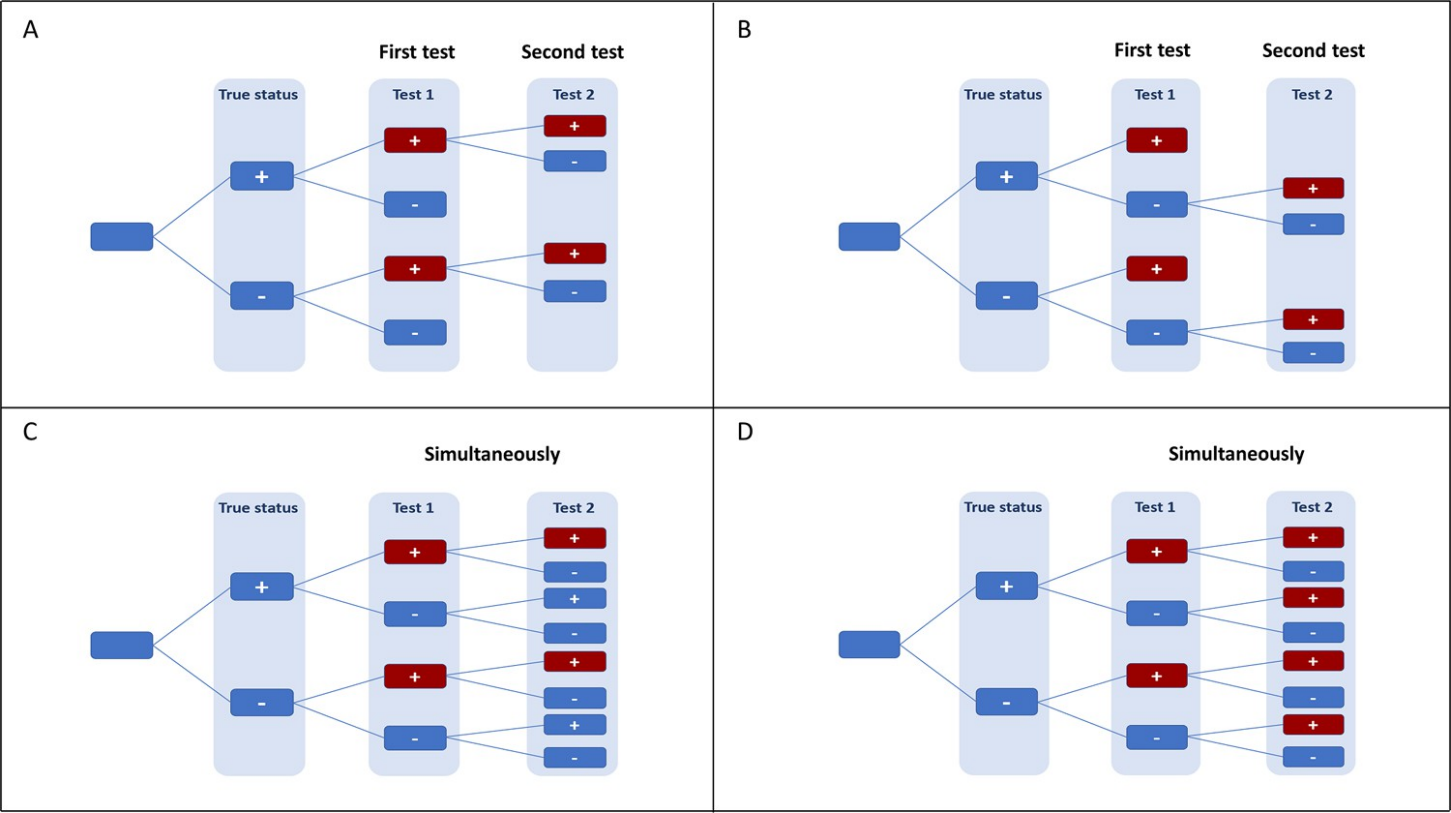
Testing strategies. A. Confirmatory testing for positives, meaning every positive test triggers a follow up test. B. Confirmatory testing for negatives, meaning that every negative triggers a follow-up test. C. Simultaneous testing, meaning that both tests are performed at the same time and the patient is considered to have a positive result if both are positive. D. Simultaneous testing, meaning that both tests are performed at the same time, and if either test is positive the patient is considered to have a positive result.

Tests are used sequentially, one after the other, with the second test used to confirm the result of the first test. There are two strategies that can be adopted:

Strategy 1: Confirmatory testing for positive results. When performing confirmatory testing for positive results, the second test is applied only if the first test is positive. This approach optimises the specificity of combination testing at the expense of sensitivity, meaning false positives are minimised (Fig 1A). This approach has been widely used to confirm diagnoses of HIV, HBV and HCV [4, 5].

#### Strategy 2: Confirmatory testing for negative results

When performing confirmatory testing for negative results, the second test is applied only if the first test is negative. This approach optimises the sensitivity of combination testing at the expense of specificity, meaning false negatives are minimised (Fig 1B). An example of this approach is the application of the Cepheid GeneXpert^®^ nucleic acid amplification test during tuberculosis screening as a follow-up test for suspected cases who return a negative sputum microscopy result [16].

### Simultaneous testing

During simultaneous testing, both tests are applied at the same time and the results are interpreted together. There are two possible approaches for the interpretation of test results under simultaneous testing.

#### Strategy 3

If both test results are positive, then the case is considered to be positive for the infection. This approach optimises the specificity of combination testing at the expense of sensitivity, meaning false positives are minimised (Fig 1C).

#### Strategy 4

If one of the test results is positive, then the case is considered to be positive for the infection. This approach optimises the sensitivity of combination testing at the expense of specificity, meaning false negatives are minimised (Fig 1D).

The average time to results refers to the average turnaround time for a testing strategy, from the time of sample collection to the time of reporting of results to the patient from whom the sample was collected.

### Calculation of combined sensitivities and specificities of two-test algorithms

Here, we describe the combined sensitivity and specificity of a two-test algorithm when the application of a confirmatory second test occurs based on either a positive or negative result of the prior test.

The combined sensitivity and specificity of two tests with individual test sensitivities of *k*_*se*1_ and *k*_*se*2_, and respective specificities of *k*_*sp*1_ and *k*_*sp*2_ when applied sequentially, with the second test applied as a confirmatory test for patients who test positive by the first test is represented by *SE*_*T*1_ and *SP*_*T*1_ and with the second test applied as a confirmatory test for patients who test negative by the first test is represented by *SE*_*T*2_ and *SP*_*T*2_, are as follows:

$$SE_{T1}=k_{se1}*k_{se2}$$

$$SP_{T1}=1-(1-k_{sp1})(1-k_{sp2})$$

$$SE_{T2}=1-(1-k_{se1})(1-k_{se2})$$

$$SP_{T2}=k_{sp1}*k_{sp2}$$

The changes in sensitivities and specificities with each of the testing strategies with corresponding changes in specificities and sensitivities compared with singular testing by either of the tests are supplied in the S1 Text. We discuss the impact on the combined accuracies with examples in the Results section. The calculations of test outcomes of true positives, false negatives, true negatives and false negatives for each of the testing approaches are provided in the S1 Text.

It is important to note that confirmatory testing for positives using a sequential testing strategy (Strategy 1) and simultaneous testing considering a case to be positive if both test results are positive for the infection (Strategy 3) predict the same sensitivities and specificities and thus the same diagnostic outcomes (true and false positives and true and false negatives). Similarly, confirmatory testing for negatives using a sequential testing strategy (Strategy 2) and simultaneous testing considering a case to be positive if either one of the test results is positive (Strategy 4) predict the same sensitivities and specificities. Nevertheless, sequential testing strategies are preferred to simultaneous testing due to having lower turnaround times and fewer resource requirements. The equations for calculating turnaround times and second test volumes are included in the S1 Text.

### Conditional dependence between tests

Conditional dependence between test results exists when the agreement of results between two tests differs from those that would be observed by chance alone. Unlike when tests are independent, the sensitivities and specificities of a second test differ according to whether the first test result was positive or negative.

If there is positive dependence of one test on the other:

1. The sensitivity of the second test among positives from the first test increases.
2. The sensitivity of the second test among negatives from the first test decreases.

The opposite is true for negative dependence. The influence of test dependence on the outcome of a combination of tests is adopted from a previously described method [17] and is described in detail in the S1 Text.

### Application of tests

#### Assumptions

We make the following assumptions in our model:

1. The two tests used in combination were independent of each other, and the likelihood of a positive test was not dependent on the result of another test. The relaxation of this assumption is explored in the S1 Text.
2. The likelihood that an individual tested positive for a disease depended on the test characteristics and disease status, irrespective of the stage of disease and corresponding viral load, presence of symptoms, and status of the host immune response.

### Model

We used a simulation approach using R statistical software, version 3.6.2 [18] to compare the outcome of testing strategies when each of the tests was applied singularly or in sequential combination, with further testing occurring for patients with positive or negative prior test results. The model accounted for two tests for COVID-19, with previously defined sensitivity and specificity and turnaround times for results. The code to run the model and generate outputs is available at https://github.com/sunildrp/covid-testing-algo.

### Scenarios

We used two hypothetical examples of use case scenarios.

1. A high infection prevalence of 20,000/100,000 (20%) in the tested population. The high prevalence scenario represents hospital settings where individuals often present with symptomatic infection, and the primary aim of testing is to obtain a diagnosis of the disease for clinical management and isolation of cases. Available literature suggests a highly variable prevalence of COVID-19 infection in healthcare settings (3–71%, median 21%) [19].
2. A low infection prevalence of 50/100,000 (0.05%) in the tested population. The low infection prevalence scenario represents a situation where the tested individuals are usually asymptomatic and tests are used to screen for COVID-19, e.g. rapid mass testing of asymptomatic individuals in England [20] and point-of-entry or border screening programmes.

### Data

The tests used for the simulation with their corresponding accuracy values are shown in Table 1. The turnaround times from the time of sample collection to result using Ag-RDT and RT-PCR were assumed to be 30 minutes and 24 hours, respectively. Ag-RDTs are performed at the site of sample collection and produce results within 15–30 minutes [21]. For RT-PCR, samples must be transported to a laboratory, and it usually takes 24 hours or more for the results depending on the testing situation.

**Table 1:** Model inputs: Sensitivities and specificities of COVID-19 tests.

	Test	Sensitivity (%)	Specificity (%)	Source
1.	Ag-RDT	70	97	*Assumption
2.	RT-PCR	90	98	Systematic review and meta-analysis of nucleic acid amplification tests on respiratory tract samples [22]

*The available sensitivity and specificity values of Ag-RDTs are measured relative to RT-PCR, with no direct information about their true performance in the population; the values vary widely across studies [8, 10, 23]. The input parameters for this analysis were empirically selected based on the available knowledge to reflect their true performance in the population. In the web-application, for pragmatic reasons, sensitivity and specificity of RT-PCR are pre-set to 100% to reflect the relationship between RT-PCR and Ag-RDTs and ease the comparison between two Ag-RDTs where the available test performance data are generated using RT-PCR as the reference standard.

### Outputs

We calculated the true positives, true negatives, false positives, and false negatives for each of the single tests and for the sequential algorithms of two tests where further tests were applied to patients who were positive or negative for prior tests. We further calculated the sensitivity and specificity for the combinations of tests, the numbers of each test required and the average turnaround times for results.

### Web-based application

We applied the same methods to develop a web-based application using R [18] and an interactive web-based interface using the R “Shiny” package [24]. The web application takes the population size being tested, infection prevalence in the tested population, diagnostic test accuracies and turnaround times for each test as inputs and calculates the test outcomes, the numbers of each test needed and the average turnaround times for each singular test and the two-test algorithms. The application also allows users to explore the impact of conditional dependence between the tests if it exists, given the availability of reliable data. The Foundation for Innovative New Diagnostics (FIND) and other diagnostics partners are already using the tool to provide technical assistance to ministries of health around the world. Feedback from early users of the tool, obtained during the provision of technical assistance to the government of Somalia, has been used to improve subsequent iterations of the tool. Virtual meetings were organized to train the early users (including JAS, CE, AT, SIM, SCA, KK, MDMF) of the web application and subsequent feedbacks were sought through virtual meetings and email correspondences. Revisions were made in the design of the interface to enhance user friendliness and outputs of the tools were modified to meet the need of the users.

## Results

### Sensitivity and specificity of two-test algorithms

Sensitivities and specificities of two-test algorithms are unaffected by disease prevalence and are thus applicable in both high- and low-prevalence scenarios. Also, the sensitivities and specificities of the two-test algorithms are the same irrespective of the order in which the tests are performed. That is to say, the algorithm of an Ag-RDT followed by RT-PCR and that of RT-PCR followed by an Ag-RDT have the same diagnostic performance. In practice, however, the cheaper and easier test would likely be chosen as the primary test, rather than the more resource intensive test. As an Ag-RDT is the preferred first test followed by confirmatory testing with RT-PCR, we focussed on the algorithm comprising an Ag-RDT followed by RT-PCR testing.

The sensitivity and specificity of the algorithm when RT-PCR is performed on patients who received a negative Ag-RDT result are 97% and 95.06%, respectively (Fig 3). By using this approach to maximise the sensitivity of testing, there is a gain in sensitivity of 27% compared with that of the Ag-RDT alone, with a loss of specificity of 1.94%. Compared with that of the RT-PCR test alone, there is a gain in sensitivity of 7% and a loss of specificity of 2.94%.

**Fig 2:**
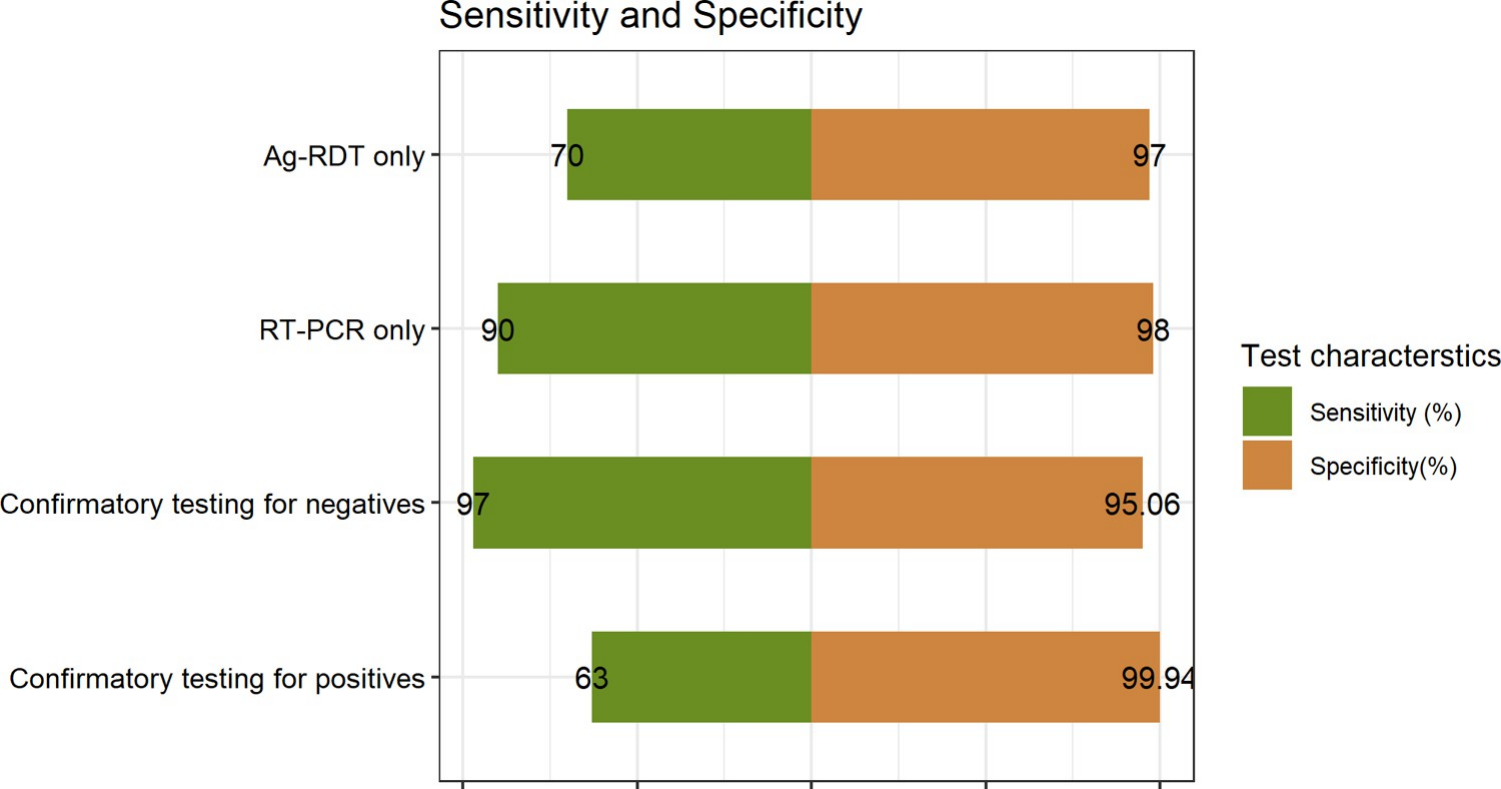
Sensitivity and specificity of combination testing compared with singular testing. The sensitivities and specificities of two-test algorithms are independent of the order of testing.

The sensitivity and specificity of the algorithm when RT-PCR is performed on patients who received a positive Ag-RDT result are 63% and 99.94%, respectively. With this approach to maximise the specificity of testing, there is a gain in specificity of 2.94% compared with that of the Ag-RDT alone, with a loss of sensitivity of 7%. Compared with that of the RT-PCR test alone, there is a gain in specificity of 1.94% and a loss of sensitivity of 27% (Fig 2).

### Test outcomes in high- and low-prevalence scenarios

We simulated the application of tests as standalone diagnostics or in two-test algorithms in 100,000 individuals and predicted the test outcomes for hospital use-cases with a high prevalence of infection and public health screening use-cases with a low prevalence of infection.

#### Hospital use-case with a high prevalence of infection

**Fig 3:**
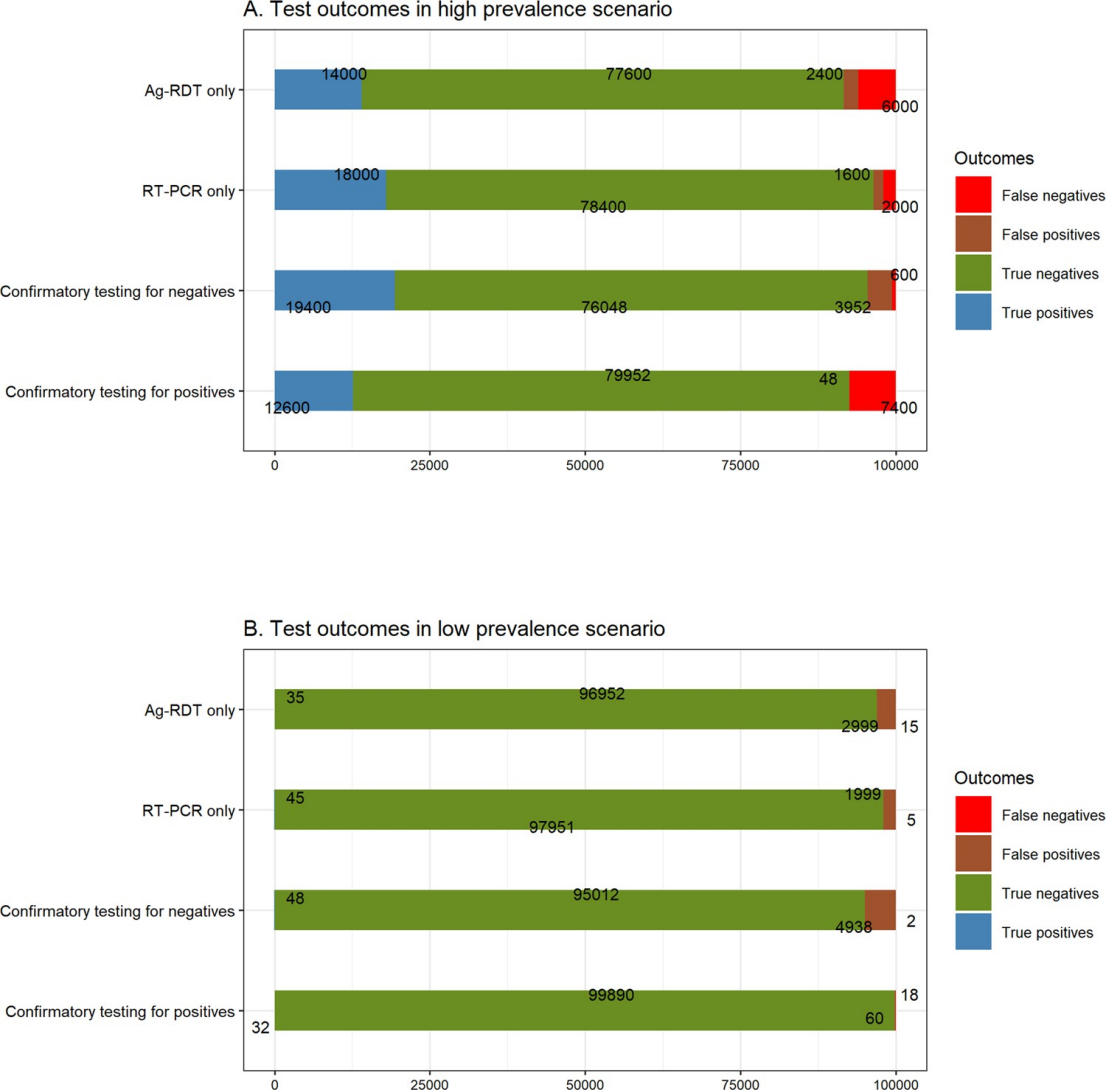
Test outcomes by testing strategy in (A) a high-prevalence scenario and (B) a low-prevalence scenario.

The aim of testing in hospital settings is to confirm the diagnosis of suspected cases, optimise the correct identification of cases, and minimise false-positive results. The application of an Ag-RDT or RT-PCR as standalone tests in a population of 100,000 with 20,000 infected individuals (infection prevalence of 20,000/100,000, or 20%) resulted in the correct identification of 14,000 and 18,000 individuals with infections, respectively, with corresponding false-positive results of 2,400 individuals using Ag-RDT alone and 1,600 individuals using RT-PCR alone. The application of both tests in the two-test algorithm, when RT-PCR was applied as a confirmatory test in patients who tested positive by Ag-RDT, resulted in the correct identification of 12,600 individuals and missed 7,400 individuals with infection. This approach minimised the false-positive results to 48 individuals (Fig 3A). In contrast, application of RT-PCR in patients who tested negative by Ag-RDT resulted in the correct identification of 19400 individuals with infection, but generated false positive results in 3952 in patients without infection. The population undergoing testing in a day, or a week may be much smaller in an actual hospital setting, but the trade-off between the correct identification of cases (true positives) and false positives holds a similar relationship, where the application of RT-PCR as a confirmatory test for individuals who test positive by Ag-RDT can largely minimise false-positive results, and prevent unnecessary treatment and distress among patients without infection. However, this should be cautiously weighted with the impact on community transmission with high number of missed cases.

#### Public health screening use-case with a low prevalence of infection

The low-prevalence scenario represents a public health screening use-case, for example, border screening programmes, where the aim of testing is to maximise the identification of individuals with asymptomatic infections and minimise false-negative results to reduce the importation of cases and subsequent transmission of infection. The application of an Ag-RDT or RT-PCR as standalone screening tests in a population of 100,000 with 50 infected individuals (infection prevalence of 50/100000, or 0.05%) resulted in the correct identification of 35 and 45 individuals with infections, respectively, with corresponding false-negative results of 15 and 5 individuals, and false positives of 2999 and 1999 individuals, respectively. The application of both tests in the two-test algorithm, when RT-PCR was applied as a second screening test in patients who tested negative by Ag-RDT, resulted in the correct identification of 48 individuals with infection, reducing the false-negative results to 2 individuals (Fig 3B). This approach however generated false positive results in 4938 individuals without infections. In contrast, application of RT-PCR in individuals who tested positive with Ag-RDT resulted in correct identification of 32 individuals with infection and produced false negative and false positive results in 18 and 60, individuals, respectively.

### Implications for test logistics and turnaround times for results

**Fig 4:**
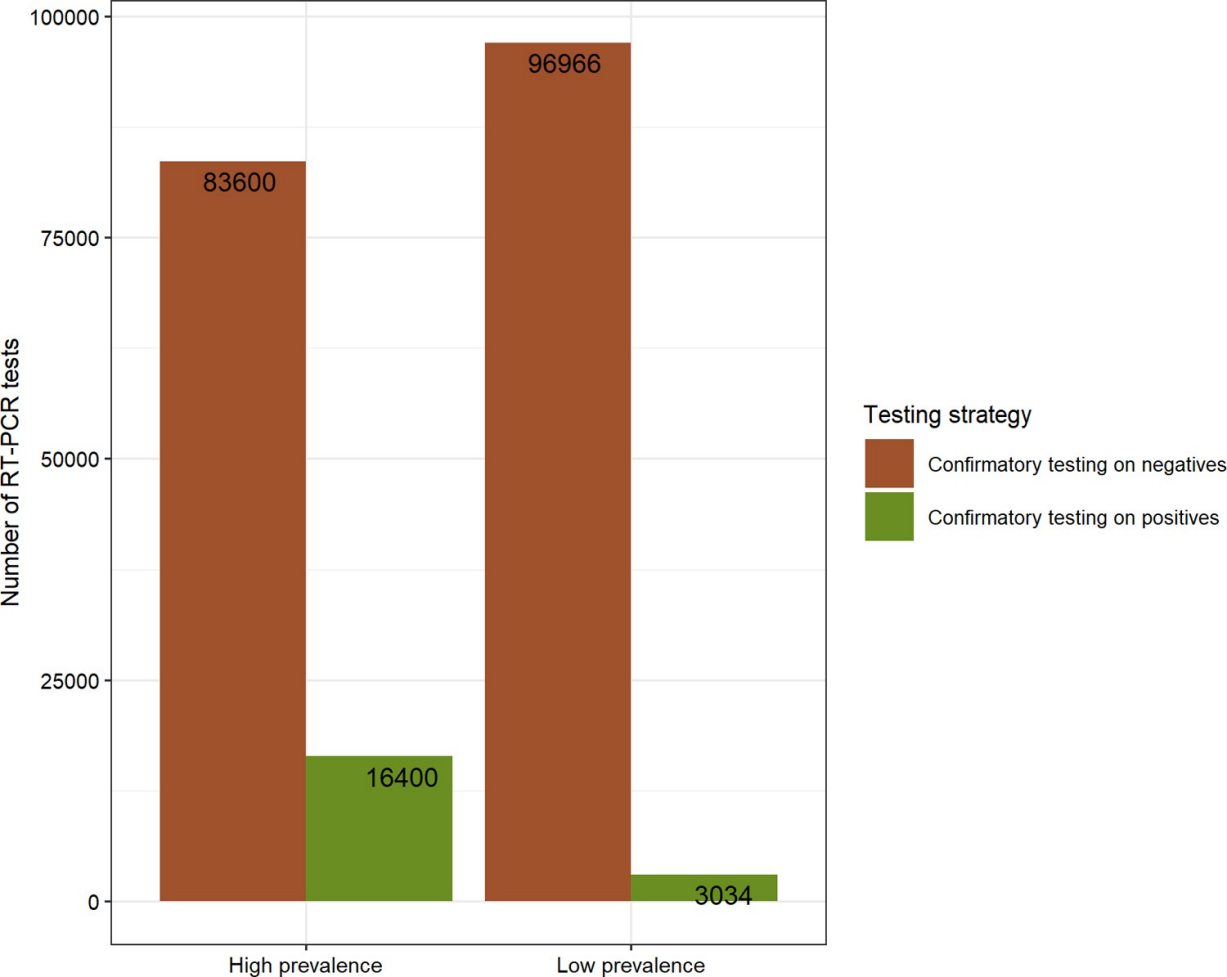
Number of RT-PCR tests. The required quantity of RT-PCR tests when applied as a second test in the two-test algorithm differed by testing strategy and infection prevalence. It corresponds to the population being tested (100,000) when used as a single test.

**Fig 5:**
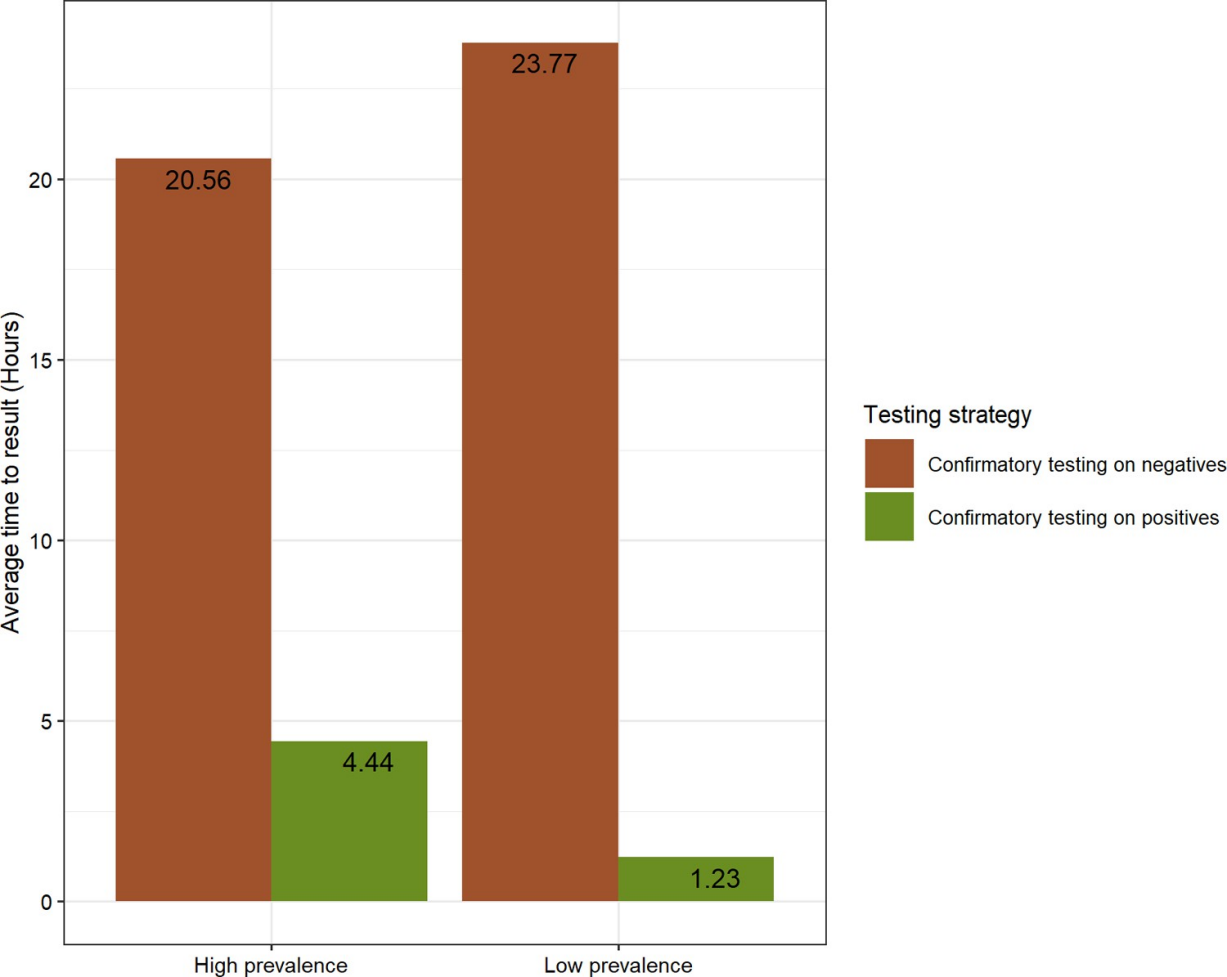
Average time taken to receive results (hours), based on assumed turnaround times of individual tests. The turnaround time for a testing strategy is the duration from sample collection to reporting of results to the patient from whom the samples were collected.

**Fig 6:**
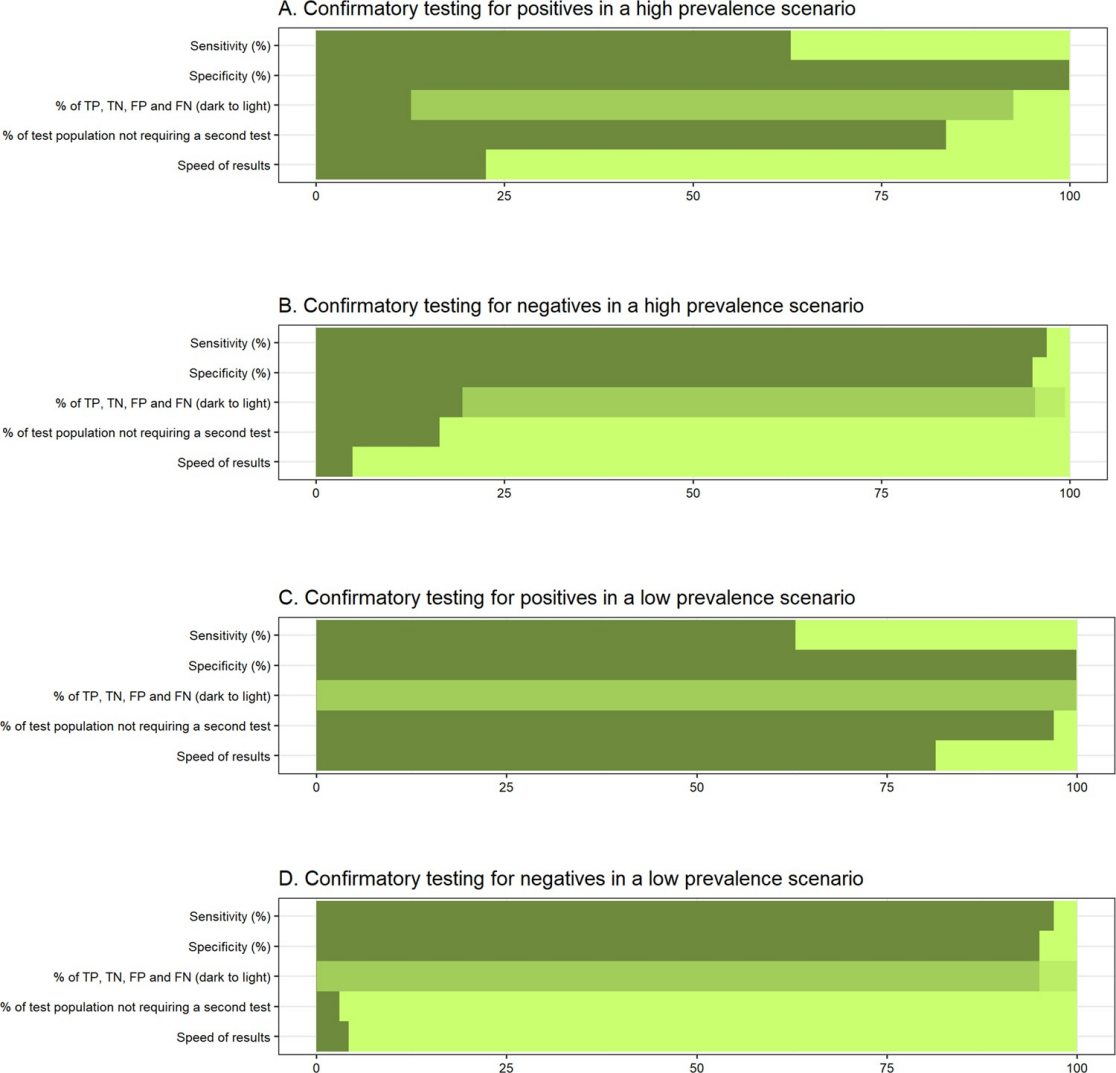
**Bar diagrams showing the assessment of test algorithms in various scenarios.** A. Confirmatory testing for positives in a high prevalence scenario. B. Confirmatory testing for negatives in a high prevalence scenario. C. Confirmatory testing for positives in a low prevalence scenario. D. Confirmatory testing for negatives in a low prevalence scenario. Interpretation: the darker the graph, the better the algorithm; TP = true positive, TN = true negative, FP = false positive, FN = false negative; the percentage of the test population who do not require a second test is a proxy for the cost-effectiveness of the algorithm; speed of results = number of tests per 100 people per hour i.e., 100/average time to results.

The predicted number of second tests required and the turnaround times for results for the algorithm involving an Ag-RDT followed by RT-PCR are shown in Figs 5 and 6, respectively. Noting that the number of Ag-RDTs would be equal to the total population size being tested, the different number of RT-PCR tests required to carry out different testing strategies is the major impact on cost and access to testing. For example, demand for RT-PCR tests when applied as a confirmatory test in patients who test positive by Ag-RDT is limited to 16,400 and 3,034 tests in the high- and low-prevalence scenarios, respectively (Fig 4), rather than 100,000 tests from each scenario when it was used as a standalone test. For the other side, only a small decrease in the number of RT-PCR tests would be observed if this test were needed to confirm negative results.

A substantial reduction in the average turnaround time for results was observed when RT-PCR was applied as a confirmatory test in patients who tested positive by Ag-RDT, i.e., 4.44 and 1.23 hours in the high- and low-prevalence scenarios, respectively, compared with 24 hours when the RT-PCR test was used alone (Fig 5).However, when applied the other way around to confirm negative results, only a small decrease in the number of RT-PCR tests required was observed.

The impact of sensitivity, specificity, test results, percentage of the population not needing a second test (based on the second test’s requirements), and speed of test results (based on the average time to receive results) for each algorithm–scenario pair on choosing an appropriate algorithm is illustrated in Fig 6.

The methodology presented here has also been used as part of ongoing country consultations as part of the COVID-19 ACT-Accelerator Diagnostic Pillar to support countries with the implementation of the Ag-RDT (Box 1).

**Box 1. A real life use case, describing the utilisation of the app as part of ongoing support activities with partners in Sub-Saharan Africa.**

The example presented represents the situation in Somalia and serves only as an example to illustrate the utility of this work to support global implementation of simple tools to mitigate the impact of COVID-19.

**How to utilise the Ag-RDTs in Somalia**:

In discussions with a team from the Ministry of Health in Somalia, the application was used to demonstrate the differences in the outcomes when the RT-PCR and various Ag-RDTs were used. The specific use case the team from the MoH were aiming to address was mass testing to a “target population” as well as testing of the “general population”. The target population was defined as symptomatic people, health care workers, high risk groups in confirmed outbreaks and contacts of confirmed cases. The general population was defined as non-symptomatic people, people at schools, workplaces, religious institutions, and port of entry. Although, an algorithm of Ag-RDT and RT-PCR was found to be the most sensitive approach, alternatives had to be found as RT-PCR capacity is currently limited in Somalia. To compensate of the limited access to molecular testing capacity, the team explored the use of multiple Ag-RDTs as a pragmatic alternative. The available estimates on the diagnostic performance characteristics of two Ag-RDTs were used to compare the possible testing strategies, combining the tests in a sequential order (SD Biosensor–SD Biosensor, SD Biosensor–Panbio, Panbio–Panbio). To allow a simple and pragmatic assessment of the times the testing strategy will give the wrong results the team established a simple error rate—measure. This was defined as the percentage of false diagnoses, either false positives or false negatives, out of the total population undergoing testing. These error rates together with positive and negative predictive values were used as the key outcomes. The app was used to calculate the aforementioned values and output was compared by the team to inform the testing strategy and its pros and cons in the local context. This iterative, data driven approach ensured that informed decisions could be taken and recommendation on the choice of algorithms were made based on the outcomes and error rate as well as final test availability. Even if the best options (RT-PCR) were limited to the team in Somalia, the simple web-application presented here allowed the team to make informed decisions and be aware of the bottle necks of their strategies. Disclosure of this consultation in scientific publication has been done in accordance with approval from the MoH in Somalia.

## Discussion

To better understand the application of multiple available tests in the diagnosis of and screening for a disease, this work explored strategies for combining tests in various use cases. The intention was to assist health authorities in choosing an appropriate combination testing strategy for infectious diseases such as COVID-19, optimising strategies for high sensitivity or specificity as needed and making the best possible use of available resources.

The high-prevalence scenario is representative of testing in hospitals to confirm diagnoses of symptomatic patients and inform their clinical management. The current recommendation for diagnostic testing in hospital settings is restricted to RT-PCR in most countries [25]; however, such capacity is often limited to central laboratories [26, 27]. The prolonged sample to result time for RT-PCR can propagate the dissemination of infection, particularly if testing is highly centralised [13, 28]. Screening programmes aim to promptly identify infected, often asymptomatic individuals in community settings or limit the importation of cases across borders and isolate any infected individuals to suppress transmission [29]. The high cost and complexity of RT-PCR and the limited sensitivity of the available Ag-RDTs discourages the application of either when screening large populations.

The application of a two-test algorithm in the hospital use-cases using an Ag-RDT followed by confirmatory RT-PCR testing of positive samples substantially reduced the false-positive rates, average turnaround times for results and RT-PCR test volumes compared with these values for RT-PCR testing alone. Reduction in false positives prevents unnecessary treatment and distress among patients without infection, reduction in average turnaround time benefits rapid identification of cases and curbs community transmission, and reduction in RT-PCR testing requirement has great value in optimizing scarce resources, especially in low resource settings. Nevertheless, given the negative impact of such a combination strategy on the sensitivity of testing, this approach increases false negative rate with potential impact on community transmission due to missed cases. Any negative test results should be interpreted with care, taking into consideration the pre-test probability of disease [8, 30]. As there exists an association of various COVID-19 symptoms and course of illness with test results, the application of two-test algorithm cannot occur in isolation, rather, decision for testing needs to be individualized to patients and account for their clinical presentation and course of infection. Similarly, application of the tests in a confirmatory algorithm to optimise the sensitivity of screening programmes, where RT-PCR was used for samples that tested negative by Ag-RDT, increased the correct identification of cases, but at the cost of an increased number of false-positive results. A positive result with such an algorithm should be interpreted cautiously, as increased false-positive results in such a strategy can lead to the unnecessary isolation of individuals and distress among the wider population.

When formulating a testing strategy for a particular use case, we must weigh the contextual value of the sensitivity and specificity of testing against the turnaround time for results and available resources [13]. A ‘one-size fits all’ approach is inappropriate [31]. When developing context-specific optimisation, multiple tests in an algorithm should be considered, to obtain the most productive output. For example, COVID-19 presents as a syndrome, showing fever and respiratory symptoms and can often not be clinically differentiated from the spectrum of other pathogens that cause respiratory infections or undifferentiated fevers [32]. While currently all focus is on COVID-19, going forward an integrated approach to the application of diagnostics for febrile illnesses should include other prevalent aetiologies [3]. The present analysis and the associated web application allow users to combine two tests for COVID-19 but also other infectious diseases and estimate the outcomes of algorithms for a combination of tests.

Despite the utility, our study has some limitations given the assumptions made in the analysis. In the absence of reliable data, the current analysis assumes that tests used in combination were independent of each other, and the likelihood of testing positive by a test was not dependent on the result of another test, which might have missed interactions between test results. Our analysis did not account for potential differences in test performances depending on the test population (e.g. asymptomatic versus symptomatic; acute versus screening). The input data for the test accuracy estimates were obtained from independent clinical studies of symptomatic infections, yet data are emerging that suggest a lower performance of Ag-RDTs in asymptomatic populations [8]. The analysis did not incorporate the possible effects of the stage of disease, corresponding viral load, and the status of host-immune responses on the test outcomes. Nevertheless, the web application allows users to run their own analysis with a context-specific, relevant test combination (e.g. Ag-RDT and RT-PCR or two different RDTs) with more realistic input data for their testing population and incorporate test-dependence in their analysis. We did not include any economic analysis of the algorithms; rather, we focussed on the test volumes. Nevertheless, end users can directly calculate the cost of tests for their specific scenario based on output test volumes.

Available diagnostic tests against various infectious diseases, including COVID-19, have limitations in their utility as standalone diagnostics at the point-of-care. A combination of available tests, using appropriate algorithms, should be considered to enhance the utility of these tests in clinical and public health decision-making [33]. The implications of the performance characteristics of a test, their availability for use, and turnaround times for results should be weighed cautiously against the contextual priorities of use cases when determining testing strategies [34, 35]. The algorithm and the web tool we have developed will assist in making these decisions.

## Supporting information

S1 Text (DOCX)
