# Correction: Two-test algorithms for infectious disease diagnosis: Implications for COVID-19

**DOI:** 10.1371/journal.pgph.0002511

**Published:** 2023-10-11

**Authors:** Sunil Pokharel, Lisa J. White, Jilian A. Sacks, Camille Escadafal, Amy Toporowski, Sahra Isse Mohammed, Solomon Chane Abera, Kekeletso Kao, Marcela De Melo Freitas, Sabine Dittrich

In the Web-based application subsection of the Methods, there is an error in the first sentence of the first paragraph. The correct sentence is: We applied the same methods to develop a web-based application (https://finddx.shinyapps.io/covid-testing-algo/) using R [18] and an interactive web-based interface using the R “Shiny” package [24].
