# Supplementary material for: Two-test algorithms for infectious disease diagnosis: Implications for COVID-19
Source: PLOS Glob Public Health. 2022 Mar 31;2(3):e0000293. doi: 10.1371/journal.pgph.0000293 (PMC10021374; doi:10.1371/journal.pgph.0000293)
Supplement: S1 Text — (DOCX) [file pgph.0000293.s001.docx]

**Two test algorithms in infectious diseases diagnosis: implications for COVID-19**

Sunil Pokharel^1, 2^, Lisa J. White^3^, Jilian A. Sacks^2^, Camille Escadafal^2^, Amy Toporowski^2^, Sahra Isse Mohammed^4^, Solomon Chane Abera^5^, Kekeletso Kao^2^, Marcela De Melo Freitas^2^, Sabine Dittrich^1,2^

^1^Centre for Tropical Medicine and Global Health, Nuffield Department of Medicine, University of Oxford, Oxford, UK

^2^Foundation for Innovative New Diagnostics (FIND), Geneva, Switzerland

^3^Big Data Institute, Li Ka Shing Centre for Health Information and Discovery, Nuffield Department of Medicine, University of Oxford, Oxford, UK

^4^National Reference Laboratory, Ministry of Health, Mogadishu, Somalia

^5^World Health Organization Country Office in Somalia, Mogadishu, Somalia

**Supplementary contents**

1. **Sensitivity, specificity and test outcomes………………………………………….…2**
2. **Second test volume and turnaround time for result………………………………..5**
3. **Test dependence ……………………………………………………………………...6**

# Sensitivity, specificity and test outcomes

| A.  **First test Second test**  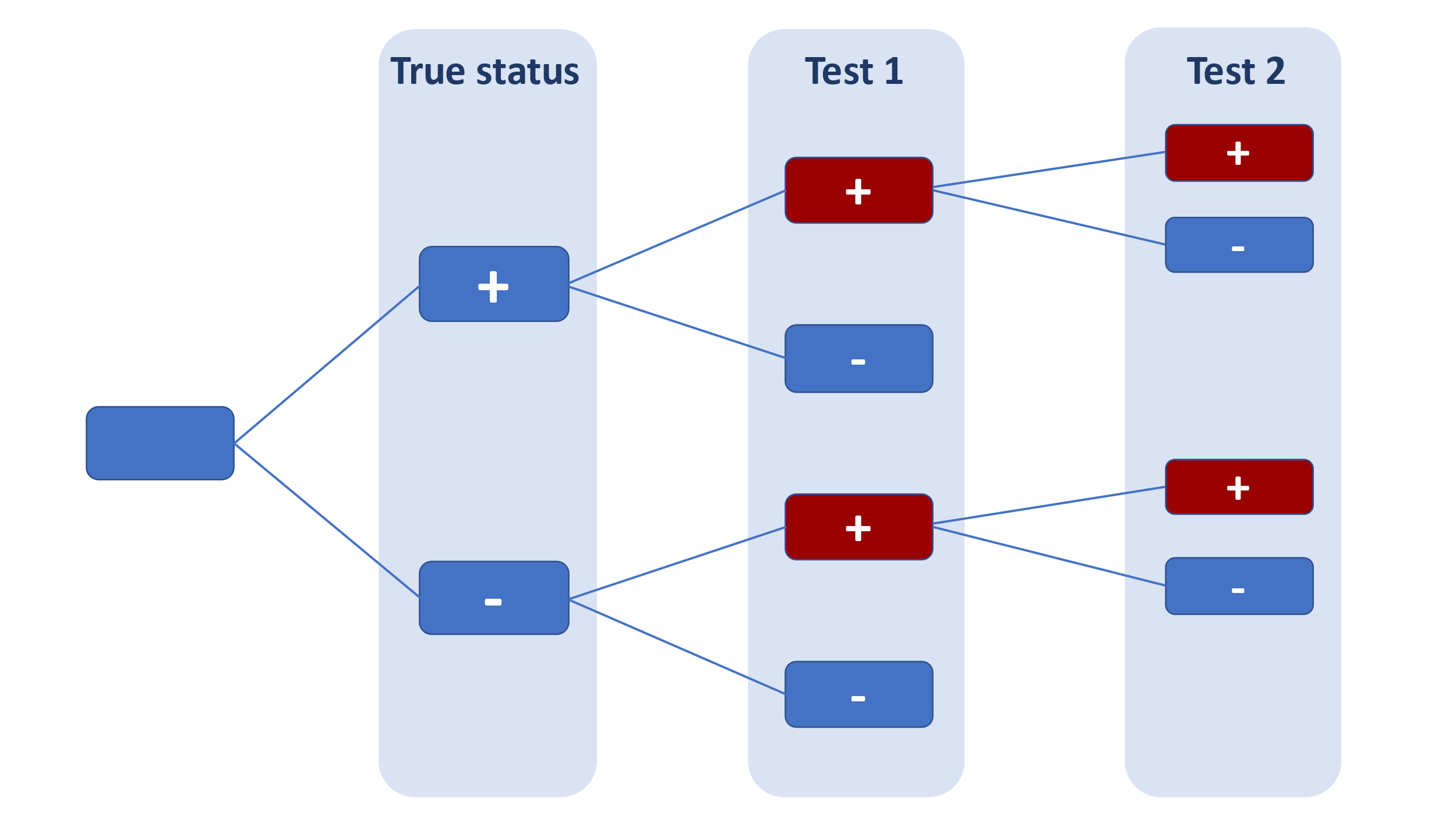 | B.  **First test Second test**  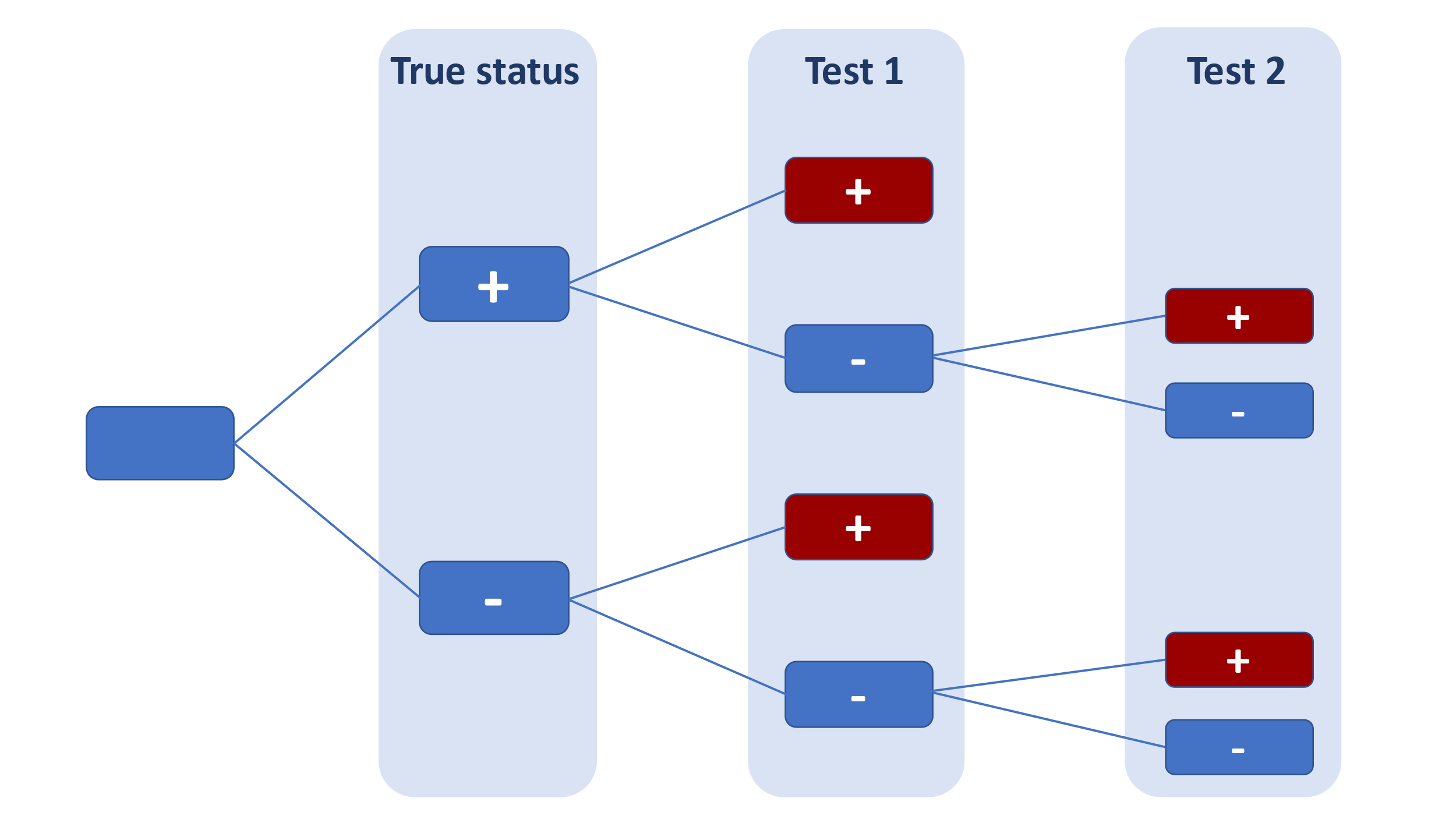 |
| --- | --- |
| C.  **Simultaneously**  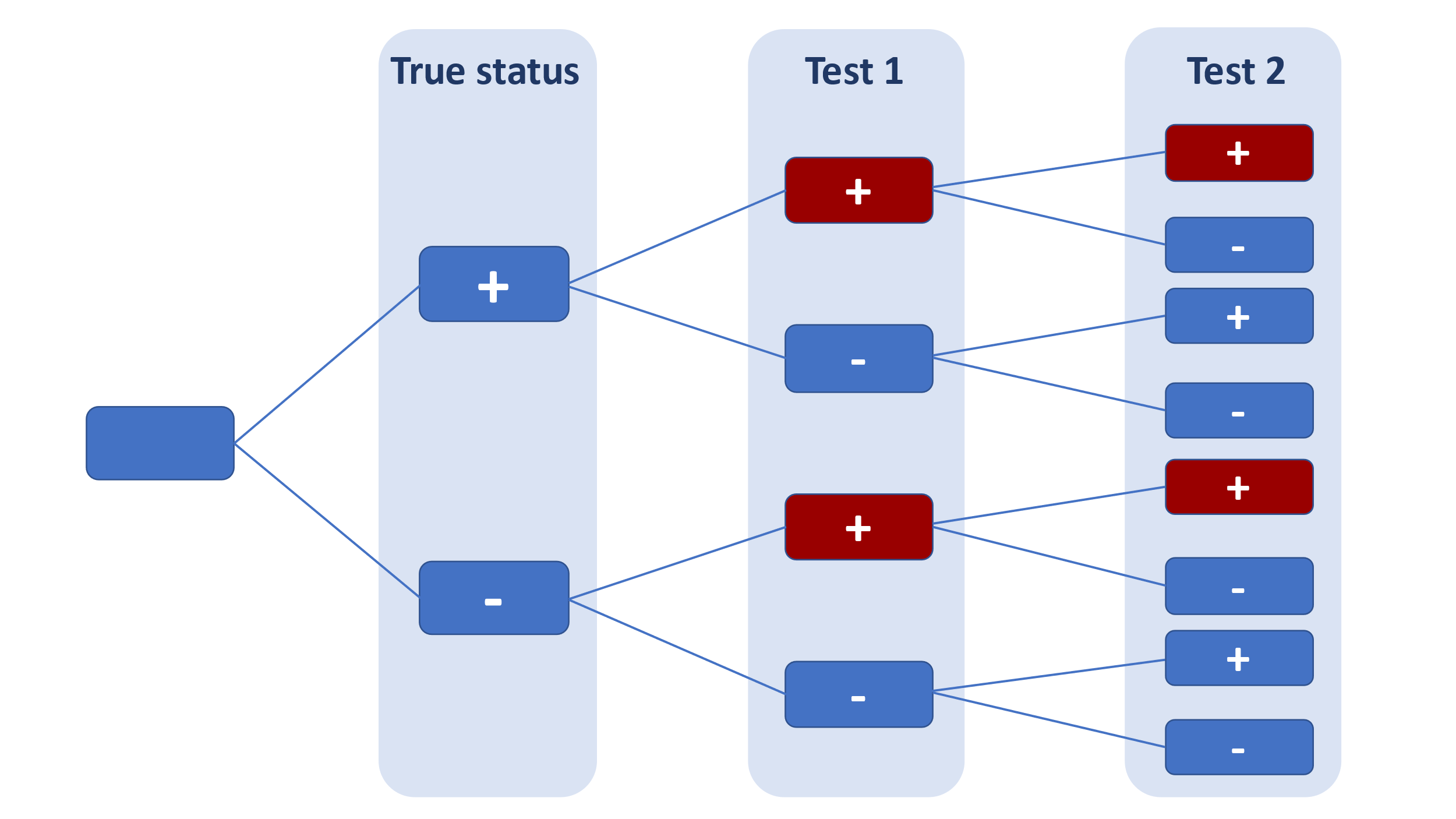 | D.  **Simultaneously**  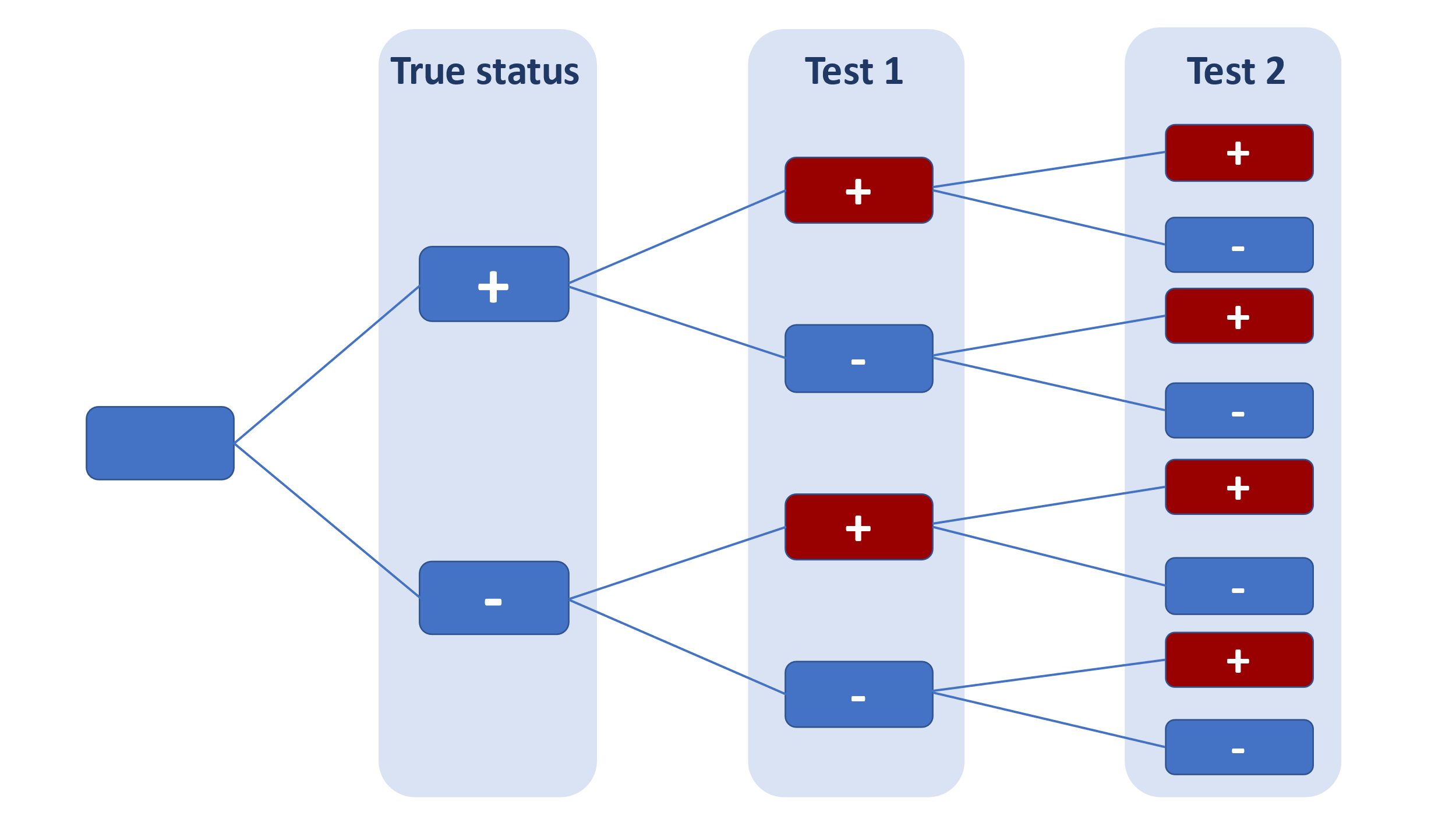 |

*Figure 1: Testing strategies; A. Confirmatory testing for positives; B. Confirmatory testing for negatives; C. Simultaneous testing and both tests positive is positive; D. Simultaneous testing and either test positive is positive.*

The combined sensitivity ($SE_{T})$ and specificity$(SP_{T})$, true positives (TP), false negatives (FN), false positives (FP) and true negatives (TN) of two tests with individual test sensitivities of $k_{se1}$ and $k_{se2}$, and respective specificities of $k_{sp1}$ and $k_{sp2}$ under the infection prevalence of *p* for different testing strategies are described below:

**1A: Sequential testing: Confirmatory testing for positives:**

Sensitivities, specificity and thus test results are equivalent to simultaneous testing and considering both testes positive as positive for the infection (1C)

$$TP=p [ 1-\left( 1-k_{se1} \right) \left( 1-k_{se2} \right)]$$

Equation 1.1

$$FN=p \left( 1-k_{se1} \right) (1-k_{se2})$$

Equation 1.2

$$FP=(1-p)(1-k_{sp1}*k_{sp2})$$

Equation 1.3

$$TN=\left( 1-p \right)*k_{sp1}*k_{sp2}$$

Equation 1.4

$$SE_{T}= 1-\left( 1-k_{se1} \right) \left( 1-k_{se2} \right)$$

Equation 1.5

$$SP_{T}= k_{sp1}*k_{sp2}$$

Equation 1.6

$$Sensitivity loss compared to test 1= k_{se1}(1-k_{se2})$$

Equation 1.71

$$Sensitivity loss compared to test 2= k_{se2}(1-k_{se1})$$

Equation 1.72

$$Specificity gain compared to test 1= k_{sp1}(1-k_{sp2})$$

Equation 1.81

$$Specificity gain compared to test 2= k_{sp2}(1-k_{sp1})$$

Equation 1.82

**1B: Sequential testing: Confirmatory testing for negatives**

Sensitivities, specificities and thus test results are equivalent to simultaneous testing and considering either of the tests positive as positive for the infection. (1D)

$$TP=p*k_{se1}*k_{se2}$$

Equation 2.1

$$FN=p (1-k_{se1}*k_{se2})$$

Equation 2.2

$$FP=\left( 1-p \right)\left( 1-k_{sp1} \right) (1-k_{sp2})$$

Equation 2.3

$$TN=\left( 1-p \right) [1-(1-k_{sp1})(1-k_{sp2})$$

Equation 2.4

$$SE_{T}= k_{se1}*k_{se2}$$

Equation 2.5

$$SP_{T}= 1-(1-k_{sp1})(1-k_{sp2})$$

Equation 2.6

$$Sensitivity gain compared to test 1= k_{se1}(1-k_{se2})$$

Equation 2.71

$$Sensitivity gain compared to test 2= k_{se2}(1-k_{se1})$$

Equation 2.72

$$Specificity loss compared to test 1= k_{sp1}(1-k_{sp2})$$

Equation 2.81

$$Specificity loss compared to test 2= k_{sp2}(1-k_{sp1})$$

Equation 2.82

**Second test volume and turnaround time for result**

The number of first tests required will be equivalent to the population under testing. The number of second test required (${2nd}_{tests}$) and average turnaround time for result ($t_{result})$ when the test is applied to the population (*P*) under different testing strategy is described below. Turnaround time for result for the first and second tests are represented by ${(t}_{result1})$and ${(t}_{result2})$ respectively.

**1A: Sequential testing: Confirmatory testing for positives**

${2nd}_{tests}$ = P (p*$k_{se1}+(1-p)(1-k_{sp1})$)

Equation 3.1

$t_{result}=${$t_{result1}*P + t_{result2}* P \left( p*k_{se1}+\left( 1-p \right)\left( 1-k_{sp1} \right) \right)\}/P$

Equation 3.2

**1A: Sequential testing: Confirmatory testing for negatives**

${2nd}_{tests}$= P (1-(p*$k_{se1}+(1-p)(1-k_{sp1})$))

Equation 3.3

$t_{result}=${$t_{result1}*P + t_{result2}* P \left( 1-(p*k_{se1}+\left( 1-p \right)\left( 1-k_{sp1} \right)) \right)\}/P$

Equation 3.4

# Conditional dependence of tests:

If there is positive dependence of one test with the other,

Sensitivity of second test among positives by first test --- increases

Sensitivity of second test among negatives by first test --- decreases

Suppose $P_{ijk}$ represents the probabilities where

i = result of 1^st^ test (1= positive, 0= negative),

j = result of 2^nd^ test (1= positive, 0= negative),

k = true infection status (1= infected, 0= non-infected)

The conditional covariance between test outcomes is represented by Y

$Y_{se}$ = conditional co-variance between tests among infected

= $P_{111}$ - $k_{se1}*k_{se2}$

Equation 4.1

$Y_{sp}$ = conditional co-variance between tests among infected

= $P_{000}$ - $k_{sp1}*k_{sp2}$

Equation 4.2

** Y > 0 if there is positive dependence and less than 0 if negative dependence

Y = 0 if independence

** $Y_{se}$ and $Y_{sp}$ can be obtained from the study data sets.

Now, applying the covariance estimates to obtain the test result with dependence,

$P_{111}$ = $k_{se1}*k_{se2}$ +$Y_{se}$

Equation 5.1

$P_{101}$ = $k_{se1}*(1-k_{se2})$ -$Y_{se}$

Equation 5.2

$P_{011}$ = $(1-k_{se1})*k_{se2}$ -$Y_{se}$

Equation 5.3

$P_{001}$ = $(1-k_{se1})*(1-k_{se2})$ +$Y_{se}$

Equation 5.4

$P_{110}$ = $(1-k_{sp1})*(1-k_{sp2})$ +$Y_{sp}$

Equation 5.5

$P_{100}$ = $(1-k_{sp1})*k_{sp2}$ -$Y_{sp}$

Equation 5.6

$P_{010}$ = $k_{sp1}*(1-k_{sp2)}$ -$Y_{sp}$

Equation 5.7

$P_{000}$ = $k_{sp1}*k_{sp2}$ +$Y_{sp}$

Equation 5.8

The effect of applying conditional dependence on the test outcomes in different testing strategies is given below:

1. **Confirmatory testing for positives:**

$$TP=p [ 1-\left( 1-k_{se1} \right) \left( 1-k_{se2} \right)- Y_{se}]$$

Equation 6.1

$$FN=p (\left( 1-k_{se1} \right) \left( 1-k_{se2} \right)+ Y_{se})$$

Equation 6.2

$$FP=(1-p)(1-k_{sp1}*k_{sp2}- Y_{sp})$$

Equation 6.3

$$TN=\left( 1-p \right)*(k_{sp1}*k_{sp2}- Y_{sp})$$

Equation 6.4

$SE_{T}= 1-\left( 1-k_{se1} \right) \left( 1-k_{se2} \right)$ - $Y_{se}$

Equation 6.5

$SP_{T}= k_{sp1}*k_{sp2}$ + $Y_{sp}$

Equation 6.6

1. **Confirmatory testing for negatives:**

$TP=p*{(k}_{se1}*k_{se2}$ + $Y_{se}$)

Equation 7.1

$$FN=p (1-k_{se1}*k_{se2}- Y_{se})$$

Equation 7.2

$$FP=\left( 1-p \right)(\left( 1-k_{sp1} \right) \left( 1-k_{sp2} \right)+ Y_{sp})$$

Equation 7.3

$$TN=\left( 1-p \right) [1-\left( 1-k_{sp1} \right)\left( 1-k_{sp2} \right)- Y_{sp})$$

Equation 7.4

$SE_{T}= k_{se1}*k_{se2}$ + $Y_{se}$

Equation 7.5

$SP_{T}= 1-(1-k_{sp1})(1-k_{sp2})$ - $Y_{sp}$

Equation 7.6
